# An Assessment of Cataract Severity Based on Antioxidant Status and Ascorbic Acid Levels in Aqueous Humor

**DOI:** 10.3390/antiox11020397

**Published:** 2022-02-16

**Authors:** Yu-Ting Tsao, Wei-Chi Wu, Kuan-Jen Chen, Chun-Fu Liu, Yi-Jen Hsueh, Chao-Min Cheng, Hung-Chi Chen

**Affiliations:** 1 Department of Ophthalmology, Chang Gung Memorial Hospital, Linkou 333423, Taiwan; sherry450047@gmail.com (Y.-T.T.); weichi666@gmail.com (W.-C.W.); cgr999@gmail.com (K.-J.C.); t6612@seed.net.tw (Y.-J.H.); 2 Department of Medicine, Chang Gung University College of Medicine, Taoyuan 33302, Taiwan; legendlcf@gmail.com; 3 Department of Ophthalmology, Chang Gung Memorial Hospital, Keelung 20401, Taiwan; 4 Program in Molecular Medicine, National Yang Ming Chiao Tung University, Taipei 112304, Taiwan; 5 Center for Tissue Engineering, Chang Gung Memorial Hospital, Linkou 33305, Taiwan; 6 Institute of Biomedical Engineering and Frontier Research Center on Fundamental and Applied Sciences of Matters, National Tsing Hua University, Hsinchu 30013, Taiwan

**Keywords:** cataract, total antioxidant capacity, ascorbic acid, cataract severity, cataract prevention

## Abstract

Cataract is the leading cause of blindness throughout the world. Currently, the cataract severity evaluation is based on the subjective LOCS III guideline. To ameliorate the evaluation system and develop an objective and quantitative analysis, we investigated the relationships among aqueous humor total antioxidant capacity (AqTAC), ascorbic acid (AqAA) concentration, and cataract severity. In this study, we enrolled 130 cataract patients who underwent phacoemulsification between April 2019 and March 2020. The AqTAC and AqAA were measured by our own developed TAC assay and commercially available kit. Cataract severity was recorded by nuclear opalescence (NO) and cortical cataract (CC) degree according to LOCS III. Cumulative dissipated energy (CDE) during phacoemulsification was recorded to verify the severity of the cataract. As a result, we found a moderate correlation between AqTAC and CDE (*p* < 0.001). In addition, we found AqTAC independently associated with the CDE when analyzed by multivariate linear regression (*p* < 0.001). AqTAC also negatively correlated to cataract severity when measured by NO and CC (*p* = 0.012 in NO grade 3 vs. grade 1; *p* = 0.012 in CC grade 2 vs. grade 1; *p* < 0.001 in CC grade 3 vs. grade 1). We further found AqAA provided 71.9 ± 13.5% of AqTAC, and showed a high correlation (rho = 0.79, *p* < 0.001). In conclusion, we found a significant correlation between AqTAC/AqAA and cataract severity measured by CDE. The correlation was superior to the correlation between LOCS III and CDE. Aqueous humor TAC owns the potential to assess cataracts in an objective and quantitative way.

## 1. Introduction

Cataract is the leading cause of blindness and afflicts millions of people annually, with a worldwide prevalence rate of 17.2% [1]. Surgical intervention remains the primary means of treatment to restore vision; however, despite the presence of novel techniques and advanced instruments for phacoemulsification treatment, complications exist [2]. Therefore, a comprehensive preoperative evaluation is important to determine treatment choices. In addition to the subjective symptom inquiry, an objective evaluation of cataract severity, which may directly affect surgical outcomes, is also necessary to provide a suitably complete investigation.

The Lens Opacities Classification System III (LOCS III) based on slit-lamp and retro-illumination photography analysis is one of the most well-known cataract severity of grading systems [3]. However, accumulative state-of-the-art evidence has emphasized the importance of ultrasonic energy expenditure during the phacoemulsification process [4,5,6,7,8]. Ultrasonic energy expenditure, which is usually recorded as cumulative dissipated energy (CDE) during the phacoemulsification process, may be used as a measure of cataract severity, and it has several advantages including the following: (1) it directly reflects lens density [9]; (2) it is an objective and quantitative value, compared with the LOCS III cataract grade classification system; (3) it is directly associated with surgical applicability and outcomes [4,5,6]. The only drawback of using CDE to measure cataract severity is that it cannot be tested before surgery. Providing a preoperative parameter that is highly correlated to CDE is highly desirable, as it may be used to guide treatment.

Oxidative stress, which is believed to be implicated in the overall process leading to the formation of cataracts, could be a potential indicator of cataract severity [10]. As the ocular lens is continually exposed to sunlight and ambient stimuli, the production of toxic free radicals is inevitable. As a result, rather than measuring oxidative status, research has focused on total antioxidant capacity (TAC) as an indicator of the capacity to confront the pathogenesis of ocular diseases [11,12,13]. Previous studies have demonstrated the protective effect of antioxidants on cataractogenesis [14,15,16]. However, most of these studies were epidemiologic or serum-based studies, and some of the results are inconsistent. The inconsistency of the results might be due to the fact that neither the epidemiologic factors nor serum factors contact with the crystalline lens directly, and therefore, they may easily be affected by methodology variations. In this regard, aqueous humor, which is in direct contact with the lens and is its primary nutritional source, may offer a better resource for exploring the correlation between aqueous humor antioxidant status and cataract formation.

**Figure 1:**
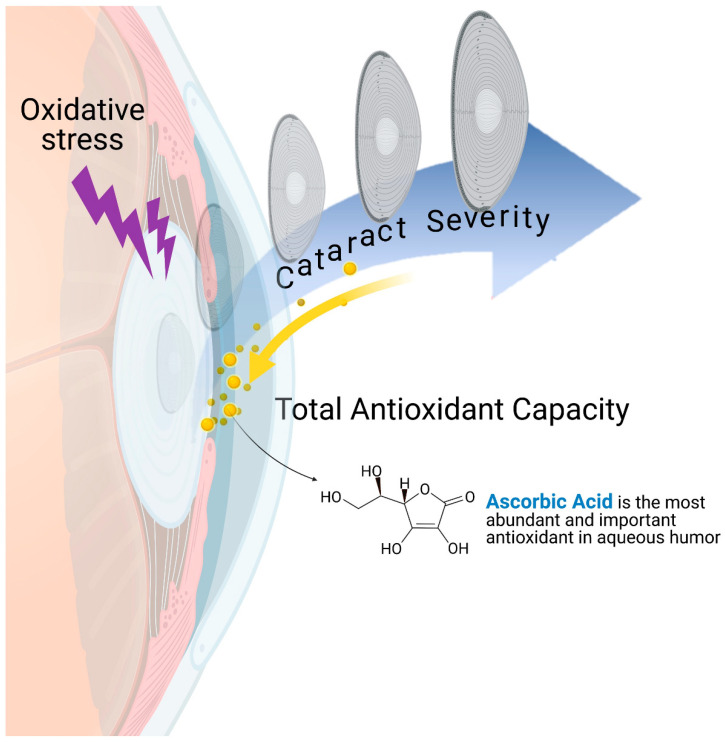
Schematic diagram illustrating the hypothetical correlations between oxidative stress, aqueous humor antioxidant capacity, and cataract severity. The ocular lens is continuously exposed to oxidative stresses such as sunlight. Oxidative injury and subsequent cataract formation are the likely results of such oxidative exposure accumulated through aging. However, aqueous humor antioxidant capacity, which is primarily composed of ascorbic acid, could postpone senility and protect the lens from cataract progression. Created with BioRender.com (accessed on 20 December 2021).

Among the elements of the antioxidant defense system in aqueous humor, ascorbic acid (AA) is a small-molecule antioxidant. AA is actively transported from serum to the aqueous humor, where it is 20–40 times more highly concentrated [17,18]. Given this, AA was believed to play a particularly noteworthy antioxidative role in aqueous humor. We hypothesized that the antioxidants in aqueous humor, especially AA, could be measured to diagnose cataract presence and severity and may also be leveraged for preventative and treatment strategies. Most of the aqueous humor-based studies to date have been hampered by insufficient sample sizes and invasive sampling techniques. Therefore, the aim of this study was to overcome these limitations and examine the associations between aqueous humor antioxidant status, AA level, and cataract severity. The primary aim of this study was to identify the correlation between TAC, AA, and CDE, and additionally to investigate the correlation between TAC, AA, and the most important cataract surgical outcomes such as best-corrected visual acuity (BCVA) improvement and intraocular pressure (IOP) change. The secondary aim was to evaluate the proportion of AA’s contribution to TAC in cataract patients and provide insight into the pathophysiological mechanisms of cataract formation. Our hypothesis is illustrated in Figure 1.

## 2. Materials and Methods

### 2.1. Study Population

All patients were recruited from the Department of Ophthalmology at Chang Gung Memorial Hospital, Linkou, Taiwan, between 1 April 2019 and 31 March 2020. The study complied strictly with the tenets of the Declaration of Helsinki and received approval from the institutional review board of Chang Gung Medical Foundation in 2019 (IRB number: 201900017B0). This was a retrospective case–control study registered in ClinicalTrials.gov (Identifiers: NCT04101591; Unique Protocol ID: 201900017B0), and written informed consents were obtained from all enrolled participants.

For sample size estimation, a pilot study with 44 subjects was conducted, and the result showed a Pearson’s correlation coefficient of 0.3 between aqueous TAC and ultrasonic energy expenditure during the phacoemulsification process. Therefore, a sample size of at least 84 samples was required in this study, to reach the statistical power of 0.8 with an α value of 0.05. As the experience and annual case volume of the surgeon have been significantly associated with cataract surgical outcomes, all patients recruited in this study received cataract surgery, preoperative evaluations, and follow-up examinations by the same experienced ophthalmologist in order to reduce technical bias [19]. The subject recruitment flow diagram for this study is presented in Figure S1. In brief, among the 240 cataract patients who received phacoemulsification and intraocular lens implantation (PHACO IOL) by one ophthalmic surgeon (Chen, H.C.), 130 were included in this study, and 103 had their IOP or BCVA checked one month later.

### 2.2. Sample Collection and Data Acquisition

Aqueous humor samples were collected from each patient at the beginning of PHACO IOL surgery. In brief, a 27-gauge needle connected to an insulin syringe was carefully inserted into the anterior chamber at the temporal limbus, and 0.1 mL of aqueous humor was aspirated. The undiluted aqueous samples were then stored immediately in a −80 °C freezer until analysis.

Patient demographics were recorded by the medical chart. Initial cataract severity assessment was graded according to the LOCS III cataract grade classification system as part of routine preoperative evaluations [3]. The degree of nuclear opalescence (NO) and cortical cataract (CC) were evaluated and recorded using a slit lamp (BQ 900, Haag-Streit, Bern, Switzerland). The final cataract severity measurement was calculated using CDE (mJ) as determined by the Infiniti Vision System (Alcon Laboratories, Inc., Fort Worth, TX, USA) following the phacoemulsification process. To evaluate surgical efficacy, the BCVA of each patient was measured using the Snellen chart, and the results were converted to the logarithm of the minimum angle of resolution (LogMAR) for analysis. For BCVA values worse than 0.01 and recorded in a semiquantitative scale, LogMAR of 1.85 was recorded for counting fingers vision, LogMAR of 2.3 was recorded for hand movement vision, LogMAR of 2.8 was recorded for light perception, and LogMAR of 3.0 was recorded for no light perception [19,20]. To evaluate surgical safety, the IOP was measured using a pneumatonometer (Canon, TX-10, Canon Corporation, Tokyo, Japan). Both BCVA and IOP were examined preoperatively and at least 1 month after cataract surgery. We then calculated the differences between preoperative and follow-up BCVA and IOP values to determine surgical efficacy and safety.

### 2.3. Aqueous Humor Antioxidant Capacity

We have developed a TAC assay based on copper (II) redox reactions [21,22]. The bicinchoninic acid (BCA) and CuSO_4_ were purchased from ThermoFisher Scientific (contained in the Pierce^TM^ BCA Protein Assay Kit, Catalog number: 23225, Waltham, MA, USA). The AA, which is a widely used antioxidant, was purchased from Fisher Scientific (B581-05, JT Baker, Phillipsburg, NJ, USA) to establish the standard curve. We established the standard curve using serial diluted 0.02–2.5 mM AA solutions. Briefly, 10 μL of the undiluted aqueous sample was applied into wells of a 96-well microplate, and 200 μL of 0.08% CuSO_4_ solution diluted by BCA was then added and incubated for 20 min away from light. The Cu^2+^ could be reduced to Cu^1+^ by various antioxidant compounds, and Cu^1+^ further formed a violet chelate complex via the interaction with BCA. The colorimetric results were measured at 570 nm by an absorbance microplate reader (Sunrise™ Tecan, Männedorf, Switzerland). The accuracy and stability of the TAC assay were verified in our previous study. The calculated limit of detection (LOD) of the TAC assay was 0.016 mM, and the limit of quantitation (LOQ) of the TAC assay was 0.053 mM. In addition, the intraassay and interassay coefficients of variability (CV) were 4.25% (*n* = 8) and 4.13% (*n* = 19), respectively. The TAC assay was stable under a wide range of pH levels (pH 4–10) [11]. We also used the colorimetric OxiSelect™ Ascorbic Acid Assay Kit (FRASC, Cell Biolabs Inc., San Diego, CA, USA) to measure aqueous humor AA concentration [23].

### 2.4. Statistical Analysis

Descriptive statistics were used for patient characterizations, which were presented as means with standard deviations or proportions as appropriate. The normality of distribution was tested for all continuous data using the Kolmogorov–Smirnov test. The proportional correlation of aqueous humor AA in TAC was presented as a pie chart and analyzed using the Spearman correlation analysis. Univariate and multivariate ordinal logistic regression analysis was used to calculate the effects of TAC on cataract severity parameters—NO and CC. The correlation between aqueous humor TAC with CDE, surgical efficacy (improvement in BCVA), and surgical safety (change in IOP) were first analyzed by Spearman’s correlation coefficient and further examined by univariate and multivariate linear regression analyses. Parameters including patient age, sampling eye site, gender, body mass index (BMI), ocular diseases, and systemic diseases were considered as confounding factors and adjusted in all the multivariate regression models. The sample size requirement and statistical power analysis of this study were calculated using the statistical program, G*Power 3.1 [24]. All other statistical analyses were conducted using Stata software version 14 (StataCorp LP, College Station, TX, USA). A *p* value of less than 0.05 was considered statistically significant.

## 3. Results

### 3.1. Study Population

A total of 130 cataract subjects were enrolled during the study period. All patients underwent examinations and sampling procedures without any adverse events. The demographics and characteristics of the 130 subjects are listed in Table 1. The mean age was 67.6 ± 8.9 years; the male patients slightly outnumbered female patients (51.5% vs. 48.5%); the mean BMI was 24.95 ± 3.49 Kg/m^2^. Additionally, 116 (89.2%) of them were diagnosed with age-related cataracts (ARCs) while the other 14 (10.8%) were diagnosed with juvenile cataracts. Age-related cataract was defined as patients who developed cataract over 55 years old, and juvenile cataract was defined as patients who developed cataract younger than 55 years old. Traumatic cataract patients were excluded, and no patient with pseudoexfoliation syndrome was enrolled.

### 3.2. Aqueous Humor Antioxidant Capacity and Ascorbic Acid Concentration

**Figure 2:**
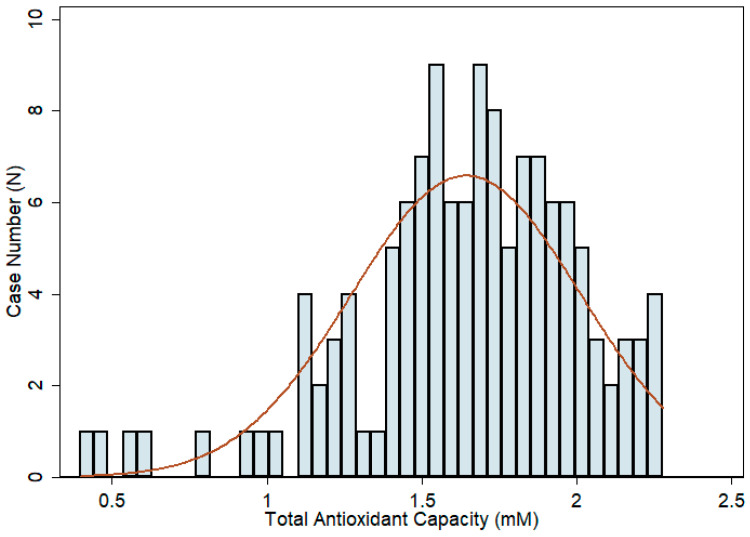
Histogram showing the distribution of aqueous humor total antioxidant capacity (TAC) in cataract patients. The data were normally distributed. The mean aqueous humor TAC was 1.643 ± 0.370 mM ascorbic acid equivalent antioxidant capacity (AAEAC) with the highest level of 2.279 mM AAEAC and the lowest level of 0.396 mM AAEAC.

**Figure 3:**
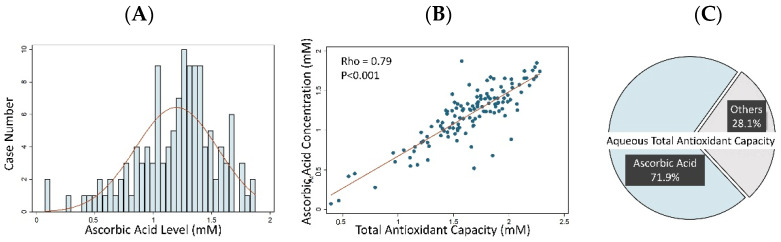
Graphs showing the aqueous humor ascorbic acid (AA) concentrations in cataract patients and its correlation to total antioxidant capacity (TAC): (**A**) histogram showing the left-skewed distribution of aqueous humor AA concentrations in cataract patients with a mean value of 1.198 ± 0.355 mM; (**B**) scatter plot showing the high correlation between aqueous humor AA concentrations and TAC with a Spearman correlation coefficient of 0.79 (*p* value < 0.001); (**C**) pie chart showing the proportional correlation between aqueous humor AA concentrations and TAC. The AA averagely contributed to 71.9% of the aqueous humor TAC in cataract patients.

Aqueous humor TAC was measured in all 130 cataract patients. All the samples showed measurable TAC results by using the TAC assay. The measured TAC values were normally distributed with a mean of 1.643 ± 0.370 mM AA equivalent antioxidant capacity (Figure 2). The concentration of aqueous humor AA was also tested in 127 patients (3 samples were not tested because of insufficient sample volumes), and the results showed non-normally distributed data, with a mean of 1.198 ± 0.355 mM (Figure 3A). There was a high positive correlation between aqueous humor TAC and AA concentrations, with a Spearman correlation coefficient value of 0.79 (*p* value < 0.001) (Figure 3B), and AA was found to be the chief antioxidant component of aqueous humor (71.9 ± 13.5%) (Figure 3C).

### 3.3. Aqueous Humor TAC, Ascorbic Acid, and Cataract Severity

**Figure 4:**
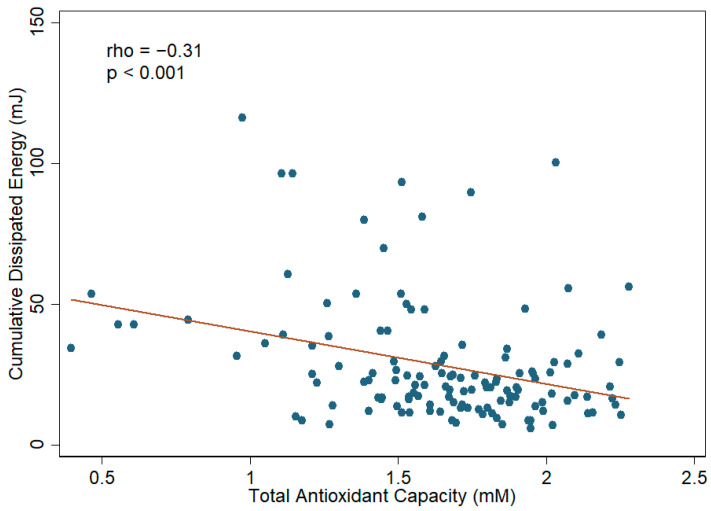
Scatter plot showing the correlation between aqueous humor total antioxidant capacity (TAC) and cumulative dissipated energy (CDE) during phacoemulsification in cataract patients. There is a medium degree of correlation between aqueous humor TAC and CDE during the phacoemulsification process in cataract patients. The Spearman correlation coefficient was −0.31 (*p* value < 0.001).

**Figure 5:**
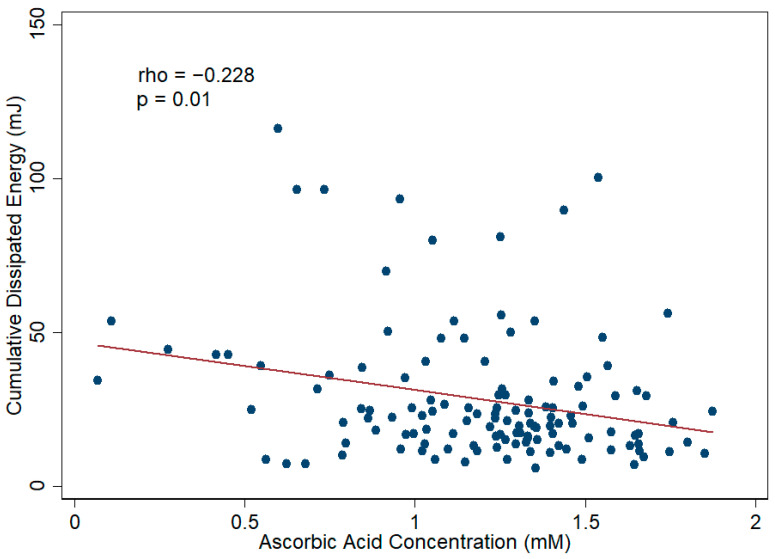
Scatter plot showing the correlation between aqueous humor ascorbic acid concentration and cumulative dissipated energy (CDE) during phacoemulsification in cataract patients. There is a medium degree of correlation between aqueous humor ascorbic acid concentration and CDE during the phacoemulsification process in cataract patients. The Spearman correlation coefficient was −0.23 (*p* value = 0.01).

Spearman’s correlation analysis revealed a statistically significant but moderate degree of negative correlation (rho = −0.31) between aqueous humor TAC and CDE (*p* value < 0.001). A scatter plot of these data is provided in Figure 4. The ascorbic acid level in aqueous humor also showed a negative correlation (rho = −0.228) with CDE (*p* value = 0.01). A scatter plot of these data is provided in Figure 5. In the subsequent linear regression analyses, the aqueous humor TAC and ascorbic acid level also revealed a statistically significant association with CDE in both univariate and multivariate models. In the multivariate linear regression analysis, the β of TAC was −18.47 with an SE of 4.89 (*p* value < 0.001), and the β of ascorbic acid was −16.27 with an SE of 5.30 (*p* value = 0.003), after adjusting all of the parameters mentioned in Table 1 including the sampling eye site, age, gender, BMI, disease diagnosis, hypertension, diabetes mellitus, and other systemic diseases. These results indicate a negative correlation between aqueous humor TAC/ascorbic acid level and CDE. The details of the linear regression models are illustrated in Table 2 and Table S1.

The correlation between TAC/ascorbic acid and CDE is superior to that of the LOCS III cataract severity, for both NO and CC, which show no significant association (*p* value > 0.05) with CDE in both the univariate and multivariate linear regression analyses (Table S2). Moreover, the associations between aqueous humor TAC and LOCS III cataract severity for NO and CC were analyzed by univariate and further multivariate ordinal logistic regression analyses. After adjusting all the confounding factors, the aqueous humor TAC was found to be negatively correlated to cataract severity. For NO grade 2 vs. grade 1, the adjusted odds ratio (OR) of aqueous humor TAC was 0.348, with confidence intervals (CI) between 0.028 and 4.301 (*p* value = 0.411); for NO grade 3 vs. grade 1, the adjusted OR was 0.078, with CI between 0.011 and 0.571 (*p* value = 0.012); for CC grade 2 vs. grade 1, the adjusted OR was 0.001, with CI between 0 and 0.355 (*p* value = 0.02); for CC grade 3 vs. grade 1, the adjusted OR was less than 0.001, with CI between 0 and 0.051 (*p* value < 0.001). More detailed data are provided in Table 3. The detailed bar chart of the aqueous humor TAC and AA in different LOCS III cataract severity is also illustrated in Figure S2.

### 3.4. Aqueous Humor TAC and Cataract Surgical Outcomes

**Figure 6:**
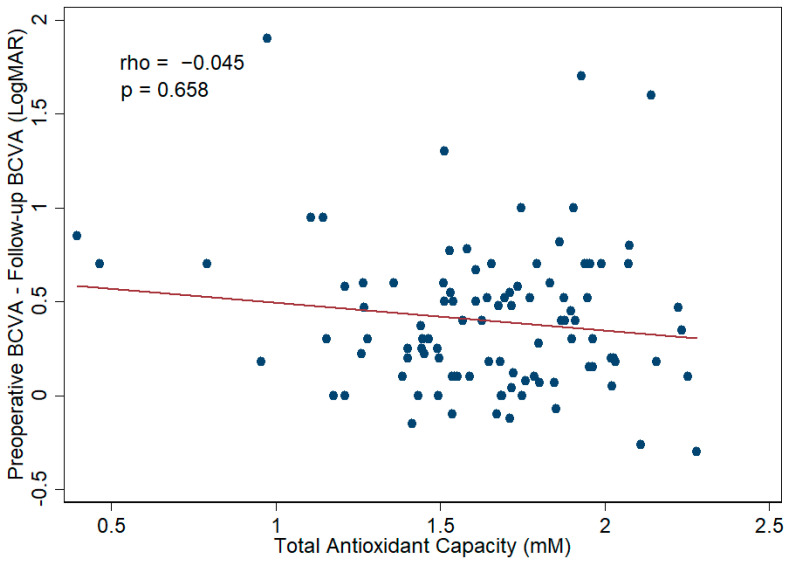
Scatter plot showing the correlation between aqueous humor total antioxidant capacity (TAC) and the best-corrected visual acuity (BCVA) improvement following cataract surgery. There was no significant correlation between aqueous humor TAC and BCVA improvement after surgery. The Spearman correlation coefficient was −0.045 (*p* value = 0.658). The BCVA was converted to the logarithm of the minimum angle of resolution (LogMAR) values for analysis.

**Figure 7:**
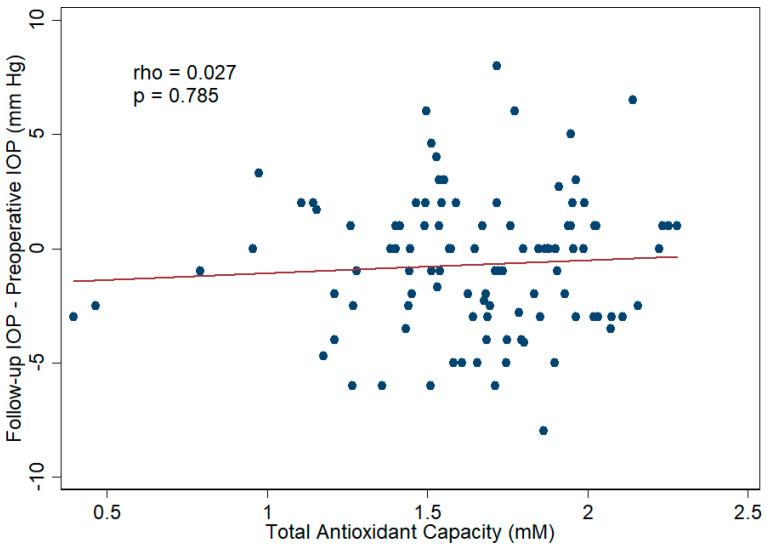
Scatter plot showing the correlation between aqueous humor total antioxidant capacity (TAC) and changes in intraocular pressure (IOP) after cataract surgery. There was no significant correlation between aqueous humor TAC and IOP changes after surgery. The Spearman correlation coefficient was 0.027 (*p* value = 0.785).

To evaluate the correlation between aqueous humor TAC and surgical efficacy, Spearman’s correlation analysis and linear regression analysis were applied; the results reveal no statistically significant association between aqueous humor TAC and BCVA improvement, with a *p* value of 0.658 according to Spearman’s correlation analysis (Figure 6), 0.188 according to the univariate linear regression analysis, and 0.335 according to the multivariate linear regression analysis. The detailed data are displayed in Table S3. Regarding the correlation between aqueous humor TAC and surgical safety, aqueous humor TAC was not significantly associated with the change in IOP according to Spearman’s correlation analysis (*p* value = 0.785, Figure 7), univariate linear regression analysis (*p* value = 0.562), and multivariate linear regression analysis (*p* value = 0.327). The detailed data are displayed in Table S4.

## 4. Discussion

This is a preliminary study to examine the proportional contribution of AA to aqueous humor TAC in cataract patients. Using the TAC assay we developed, we further investigated the correlation between aqueous humor TAC and cataract severity, surgical efficacy, and surgical safety. Most importantly, we found that aqueous humor TAC level provided a significant correlation to cataract severity, which is presented in the relation between aqueous humor TAC/AA and CDE/LOCS III cataract severity of NO and CC. Although previous studies have reported that antioxidants are associated with reduced risk of cataract development, most of them were epidemiological studies, nutrition studies, or serum-based studies, none of which involves direct contact with the lens because of the blood–ocular barrier [25,26]. To provide insight into the correlation between TAC and cataractogenesis, we chose to directly analyze aqueous humor, the primary circulatory system of the lens.

There are several ways to evaluate the TAC, including the oxygen radical absorbance capacity (ORAC) assay [27], the DPPH assay [28], the Trolox equivalent antioxidant capacity (TEAC) assay [29], the ferric reducing antioxidant power (FRAP) assay [30], the cupric reducing antioxidant capacity (CUPRAC) assay [31], etc. However, because of the complexity of TAC, which includes enzymes, proteins, and small molecules, none of the assays could completely evaluate the antioxidant capacity of all these antioxidants. Furthermore, because of the different mechanisms and targets of each assay, the assays may show incoherent results between each other [32,33]. Therefore, choosing an appropriate TAC assay that could represent the majority of the TAC in the sample is important. Table 4 demonstrates the published measuring methods to evaluate TAC in aqueous humor. Although there were obvious differences in the TAC results measured by different assays, we could find that most researchers choose to use colorimetric change based on Fe^2+^ to evaluate the TAC in aqueous humor, and their results showed much more TAC in aqueous humor than the rest. This result may be because small molecules are major components of TAC in aqueous humor, and colorimetric change based on metal ions is an appropriate method to evaluate small-molecular-type antioxidants. However, there are still several drawbacks of the current, commercially available FRAP assay. First, the relatively large sample volume requirement, and second, the requirement of low pH level to maintain the stability of Fe^3+^-TPTZ compound. Therefore, we have developed our own assay based on copper (II) redox reactions to measure the TAC in aqueous humor [11]. In our previously published paper, we developed our TAC assay to measure TAC in aqueous humor, which requires only 10 μL sample per test. In addition, it could produce results with high accuracy, insistence, and good stability among a wide range of pH levels and experimental times. We also compared the results with commercially available FRAP assay, which showed high consistency. Furthermore, we built up the standard curve by ascorbic acid, which is the most abundant antioxidant in aqueous humor. This increased the similarity between standard solution and clinical samples. Therefore, although our TAC assay still could not measure all the antioxidants in aqueous humor, we believe that our own developed TAC assay may be the most appropriate TAC assay to evaluate TAC in aqueous humor.

Our finding that aqueous humor TAC is negatively correlated with cataract severity is consistent with that of other researchers that examined cataract risk factors and possible pathological mechanisms. Existing evidence has ranked age, ultraviolet exposure, use of tobacco, and hyperglycemia as the top risk factors for cataract formation; each of these have an obvious correlation to oxidative stress [45,46,47]. In addition, Garner B et al. reported a correlation between hydroxyl radical formation in the lens and cataract severity [48]. There is also evidence that antioxidant compounds could prevent the lens from opacification and cataract formation [49,50]. An interesting findings in our study showed that there is no significant correlation between TAC/ascorbic acid and age (Figure S3). Therefore, the real correlation between TAC/ascorbic acid and cataract formation is worthy of further investigation. Our study extended previous research and found that aqueous humor TAC is significantly associated with both cataract hardness (according to CDE) and LOCS III cataract severity of NO and CC. Moreover, we found that aqueous humor TAC and ascorbic acid demonstrated a better correlation to CDE than the LOCS III cataract severity grading system. Although the LOCS III cataract severity grading system is one of the standard preoperative assessments of cataracts, the exact correlation between the grading system and the ultrasonic energy expenditure is doubtful. In a previous study, only low degrees of correlations were found between LOCS III cataract severity and CDE (R^2^ = 0.15 for NO and R^2^ = 0.18 for nuclear color, and no correlation between CC and CDE) [51], whereas in our study, no significant association was found between the two parameters. Thus, it is plausible that measuring aqueous humor TAC, which displays a medium degree of correlation with CDE, might be a better method for providing an accurate preoperative estimation, and choosing the appropriate cataract extraction strategy. For example, some aqueous humor can be aspirated when making the side pore in cataract surgery. Then, we can use our TAC assay to analyze the TAC of the aqueous humor before starting the phacoemulsification process. Then, based on the results, surgeon could tailor the cataract surgery for the patient by adjusting the parameters of phacoemulsification, choosing between the phacoemulsification process or traditional extracapsular cataract extraction, determining the use of tension ring, etc. A complete surgical plan may improve the surgical outcome and minimize the complication rate. However, the applicability of this scenario requires further clinical validation.

The superior correlation between aqueous humor TAC and cataract severity might be attributable to the objective and quantitative recording system, and the close relationship between oxidative stress and cataract formation. On the other hand, our findings revealed no significant associations between aqueous humor TAC and the surgical efficacy or surgical safety evaluated by BCVA and IOP. These results are reasonable according to previous studies showing that CDE was not associated with postoperative IOP changes [52]. However, previous studies have delineated the correlation between CDE and postoperative dry eye incidence, intraocular mechanical trauma, macular edema incidence, retinal vasculature, and perfusion density [53,54,55,56]. Moreover, there is a more significant correlation between CDE and corneal endothelial cell loss during the phacoemulsification process [6,57,58]. Although there is no evidence directly explaining the correlation between aqueous humor TAC and cataract surgical outcomes, aqueous humor TAC has the potential to be influential. As we have revealed the moderate correlation between aqueous humor TAC and CDE, the correlations between aqueous humor TAC and more aspects of clinical cataract surgical outcomes are worthy of further research. 

There are several limitations of this study. As an invasive procedure was necessary to obtain the aqueous humor sample, the recruitment of healthy subjects was prohibited, and a control group was not possible. Examining aqueous humor TAC levels repeatedly in a longitudinal follow-up setting was also not possible. To overcome these limitations and provide a trustworthy result, we designed the study in a rigorous way. First, we conducted a pilot study to calculate the sample size requirement of this study to reach a satisfactory statistical power. Second, all the patients who participated in this study received preoperative cataract severity evaluations and cataract surgery by the same ophthalmologist to reduce technical bias. Third, we measured the correlation between aqueous humor TAC and cataract severity in many ways including the LOCS III cataract severity grading system for NO and CC; CDE determined during cataract surgery was used to confirm results. Last, we adjusted the potential confounding factors in all of our regression models and found age and diabetes mellitus to be significant confounders in our multivariate regression analysis. The other limitation of this study is that we did not enroll and discuss the systemic and topical medication use of the patients, and the current findings are not sufficient enough to conclude that antioxidant therapy can prevent cataract formation or progression. Further clinical trials are warranted to verify the applicability of antioxidant therapy in slowing cataract development.

## 5. Conclusions

In summary, ascorbic acid contributed to the majority of aqueous humor TAC, and both the aqueous humor TAC and ascorbic acid demonstrated negative correlations to CDE use during cataract surgery. Moreover, aqueous humor TAC and ascorbic acid show a better correlation to cataract severity measured by CDE than the current LOCS III cataract severity grading system. However, there was no significant correlation between aqueous humor TAC and postoperative BCVA and IOP changes. According to these findings, measuring the TAC levels in aqueous humor owns the potential to provide clinicians with a useful cataract severity scoring system for the determination of cataract extraction strategy; measuring the TAC levels could also provide the cornerstone and guidance for examining potential antioxidant-based cataract therapies. Future advancements in optic devices for noninvasive assessments of aqueous humor TAC would be significantly advantageous to this prescribed process and additional processes that may be developed as a result of this guidance.

## Figures and Tables

**Table 1:** Patient characteristics (*n* = 130).

Basic Characteristics	
OD/OS, *n*	59/71
Age, mean ± SD (Years)	67.6 ± 8.9
Gender, M/F *n* (%)	67 (51.5%)/63 (48.5%)
BMI, mean ± SD (Kg/m^2^)	24.95 ± 3.49
Disease diagnosis, *n* (%)	ARC: 116 (89.2%) Juvenile cataract: 14 (10.8%)
Underlying Diseases	
Hypertension, *n* (%)	51 (39.2%)
Diabetes mellitus, *n* (%)	28 (21.5%)
Other underlying systemic diseases, *n* (%)	55 (42.3%)

ARC = age-related cataract; BMI = body mass index; F = female; M = male; OD = oculus dextrus; OS = oculus sinister; SD = standard deviation. Other systemic diseases include dyslipidemia, heart diseases, kidney diseases, lung diseases, liver diseases, stroke, immunocompromised status, and autoimmune diseases.

**Table 2:** Univariate and multivariate linear regression analyses for cumulative dissipated energy during the phacoemulsification process.

Variable	Univariate Regression			Multivariate Regression		
	B	SE	*p* Value	B	SE	*p* Value
Total antioxidant capacity	−18.62	4.85	<0.001 ***	−18.47	4.89	<0.001 ***
Basic characteristics						
OD/OS	0.31	3.79	0.934	−0.128	3.53	0.971
Age	0.45	0.21	0.034 *	0.719	0.27	0.008 **
Gender	−2.58	3.77	0.496	1.130	3.65	0.758
BMI	−0.01	0.54	0.99	−0.426	0.55	0.444
Disease diagnosis	−0.11	6.09	0.986	13.92	7.82	0.078
Underlying disease						
Hypertension	0.61	3.87	0.876	0.676	4.3	0.875
Diabetes mellitus	11.4	4.48	0.012 *	8.623	4.44	0.055
Other underlying systemic disease	−3.73	3.81	0.329	−3.996	4.03	0.323

B = beta coefficient; BMI = body mass index; OD = oculus dextrus; OS = oculus sinister; SE = standard error. Age, eye site, gender, body mass index, disease diagnosis, and underlying diseases were adjusted in the multivariate regression as confounding factors. *p* value of the multivariate regression model is 0.001. Other systemic diseases include dyslipidemia, heart diseases, kidney diseases, lung diseases, liver diseases, stroke, immunocompromised status, and autoimmune diseases. *: *p*-value < 0.05; **: *p*-value < 0.01; ***: *p*-value <0.001.

**Table 3:** Univariate and multivariate ordinal logistic regression analyses for total antioxidant capacity on cataract severity of nuclear opalescence and cortical cataract.

	Crude Odds Ratio		Adjusted Odds Ratio	
	OR (95% CI)	*p* Value	OR (95% CI)	*p* Value
Nuclear Opalescence				
Grade 2 vs. Grade 1	0.453 (0.04–5.18)	0.524	0.348 (0.028–4.301)	0.411
Grade 3 vs. Grade 1	0.11 (0.016–0.735)	0.023 *	0.078 (0.011–0.571)	0.012 *
Cortical Cataract				
Grade 2 vs. Grade 1	0.027 (0.001–1.055)	0.053	0.001 (0–0.355)	0.02 *
Grade 3 vs. Grade 1	0.005 (0–0.147)	0.002 **	< 0.001 (0–0.051)	<0.001 ***

OR: odds ratio; CI: confidence interval. Age, eye site, gender, body mass index, disease diagnosis, and underlying diseases were adjusted as the confounding factors. *: *p*-value < 0.05; **: *p*-value < 0.01; ***: *p*-value < 0.001.

**Table 4:** The published measuring methods to evaluate total antioxidant capacity in aqueous humor.

Reference	Total Antioxidant Capacity (Unit: mmol/L Trolex)	Sample Size	Measurement Method
Aksoy et al. 2001 [34]	0.41 ± 0.04	16	Spectrophotometric method (elimination of ABTS^R+^)
Mancino et al. 2011 [35]	0.94 ± 0.26	14	Fluorescence method (inhibit the peroxidation of β-phycoerythrin)
Sorkhabi et al. 2011 [12]	0.34 ± 0.15	27	Spectrophotometric method (elimination of ABTS^+^)
Beyazyıldız et al. 2013 [36]	2.54 ± 0.71	33	Colorimetric change in Fe^2+^
Nucci et al. 2013 [37]	0.963 ± 0.302	26	Fluorescence method (inhibit the peroxidation of β-phycoerythrin)
Beyazyıldız et al. 2014 [38]	2.5 ± 0.7	25	Colorimetric change in Fe^2+^
Kirboga et al. 2014 [39]	0.65 ± 0.09	22	Colorimetric method (reduction of 2,2′-azino-bis radical)
Ergan et al. 2016 [40]	1.80 ± 0.79	31	Colorimetric method (inhibit oxidation of H_2_O_2_)
Kulaksızoglu et al. 2016 [41]	0.77 ± 0.07	35	TAS kit (unknown mechanism)
Altinisik et al. 2018 [42]	0.78 ± 0.46	28	Colorimetric change in Fe^2+^
Bozkurt et al. 2019 [43]	0.39 [0.39–0.44]	28	Colorimetric immunodiagnostic assay (H_2_O_2_ elimination)
Siegfried et al. 2019 [44]	0.479 ± 0.146	72	Luminescence method (elimination of alkyl peroxyl radicals)

## Data Availability

Data is contained within the article and supplementary material.

## Supplementary Materials

The following supporting information can be downloaded at: https://www.mdpi.com/article/10.3390/antiox11020397/s1, Figure S1: Flow diagram presenting the recruitment process of the study subjects, Figure S2: Bar chart showing the total antioxidant capacity (TAC) and ascorbic acid concentration (AA) in each LOCS severity score, Figure S3: Scatter plot showing the correlation between aqueous humor total antioxidant capacity (TAC)/ascorbic acid concentration (AA) and Patient Age, Table S1: Univariate and Multivariate Linear Regression Analyses of ascorbic acid for cumulative dissipated energy during the phacoemulsification process, Table S2: Univariate and multivariate linear regression analyses for cumulative dissipated energy during the phacoemulsification process based on nuclear opalescence and cortical cataract, Table S3: Univariate and multivariate linear regression analyses for the improvement of best-corrected visual acuity after cataract surgery, Table S4: Univariate and multivariate linear regression analyses for the changes of intraocular pressure after cataract surgery.
