# Supplementary material for: An Assessment of Cataract Severity Based on Antioxidant Status and Ascorbic Acid Levels in Aqueous Humor
Source: Antioxidants (Basel). 2022 Feb 16;11(2):397. doi: 10.3390/antiox11020397 (PMC8869206; doi:10.3390/antiox11020397)
Supplement: Supplementary file 1 [file antioxidants-11-00397-s001.zip › Supplementary Figure S1-edited.pdf]

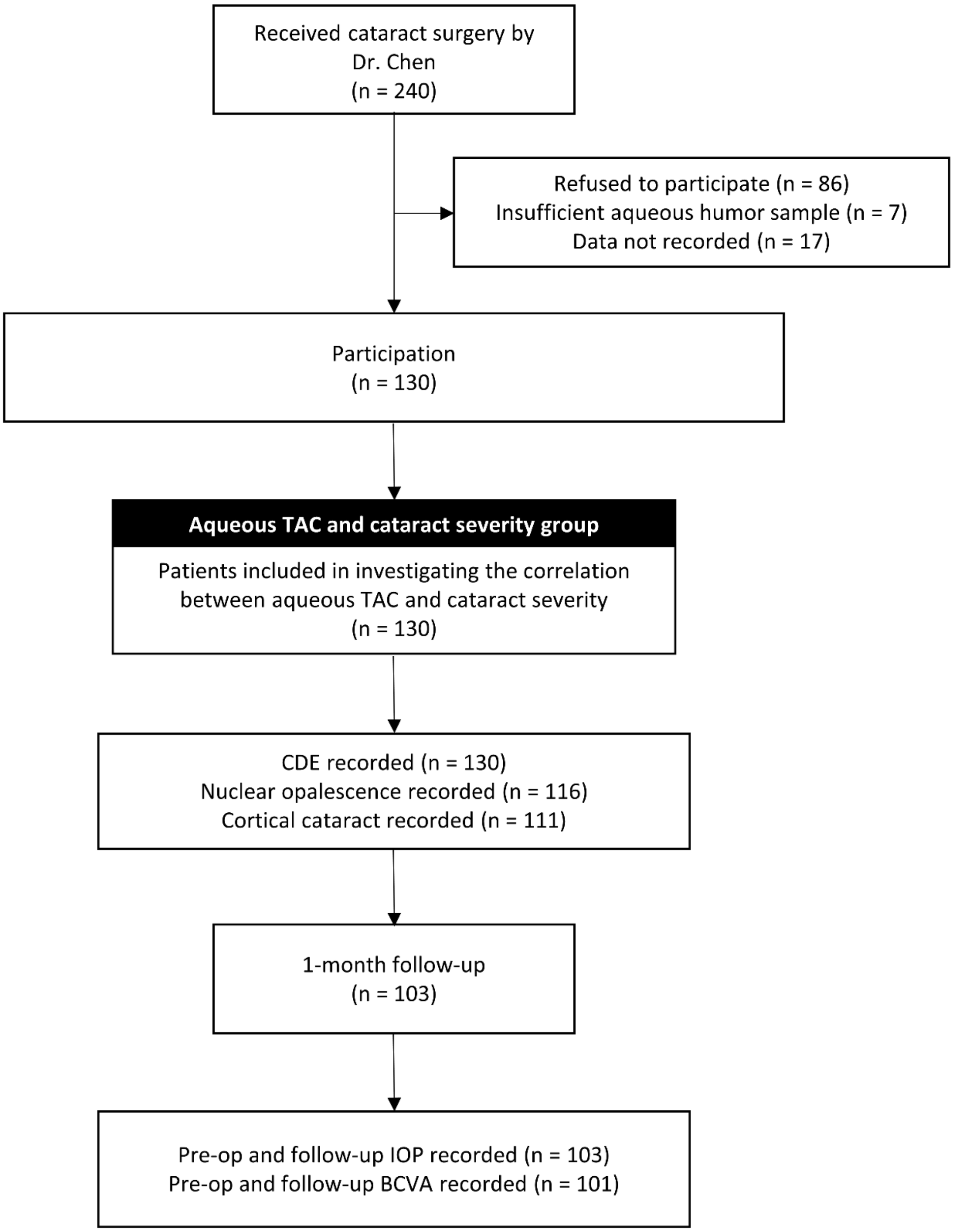


BCVA = best corrected visual acuity; CDE = cumulative dissipated energy; IOP = intraocular pressure; TAC = total antioxidant capacity

**Supplementary Figure S1.** Flow diagram of the recruitment process of the study subjects.
