# Supplementary material for: An Assessment of Cataract Severity Based on Antioxidant Status and Ascorbic Acid Levels in Aqueous Humor
Source: Antioxidants (Basel). 2022 Feb 16;11(2):397. doi: 10.3390/antiox11020397 (PMC8869206; doi:10.3390/antiox11020397)
Supplement: Supplementary file 1 [file antioxidants-11-00397-s001.zip › Supplementary Figure S2-edited.pdf]

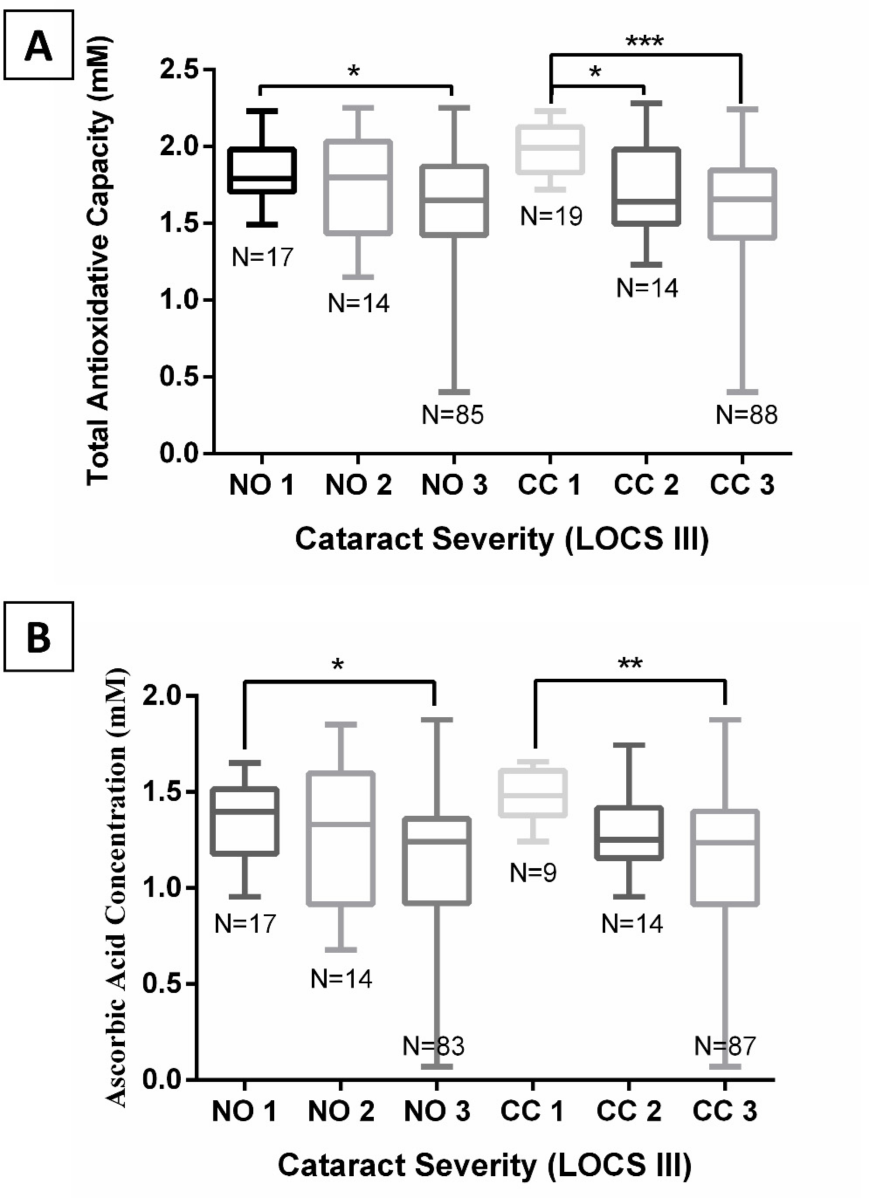

Figure S2. Bar chart showing the total antioxidant capacity (TAC) and ascorbic acid concentration (AA) in each LOCS severity score: (**A**)The bar chart of TAC in each LOCS severity score. The aqueous humor TAC shows a significant difference between nuclear opalescence (NO) grade 1 group and grade 3 group when using Mann–Whitney U test (P value = 0.016). There is also a significant difference between cortical cataract (CC) grade 1 and grade 2 groups, and grade 1 and grade 3 groups when analyzed by Mann–Whitney U test (P value = 0.039 and P value < 0.001 respectively); (**B**) the bar chart of AA in each LOCS severity score. The aqueous humor AA level is significantly different between nuclear opalescence (NO) grade 1 and grade 3 groups when analyzed by Mann–Whitney U test (P value = 0.016). There is also a significant difference between cortical cataract (CC) grade 1 and grade 3 groups when analyzed by Mann–Whitney U test (P value =0.002). *: p-value < 0.05; **: p-value < 0.01; ***: p-value <0.001.
