# Supplementary material for: An Assessment of Cataract Severity Based on Antioxidant Status and Ascorbic Acid Levels in Aqueous Humor
Source: Antioxidants (Basel). 2022 Feb 16;11(2):397. doi: 10.3390/antiox11020397 (PMC8869206; doi:10.3390/antiox11020397)
Supplement: Supplementary file 1 [file antioxidants-11-00397-s001.zip › Supplementary Figure S3-edited.pdf]

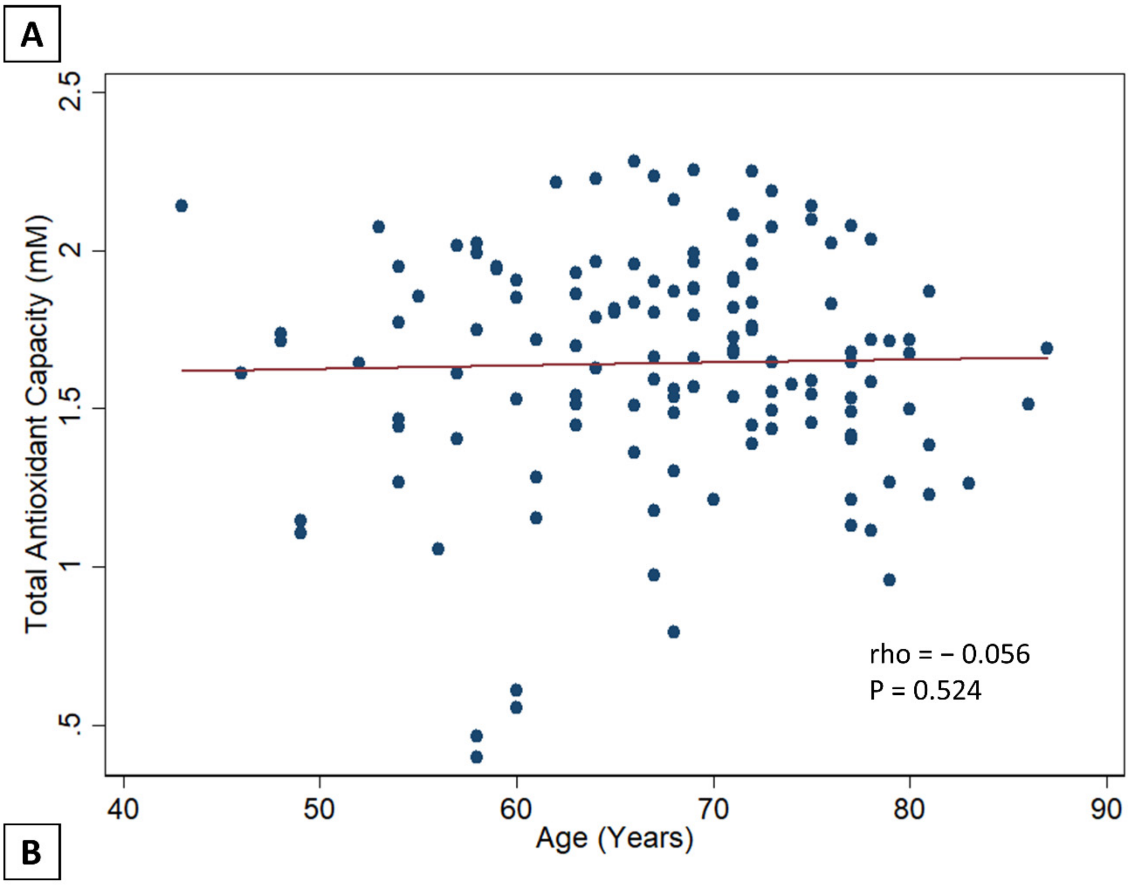

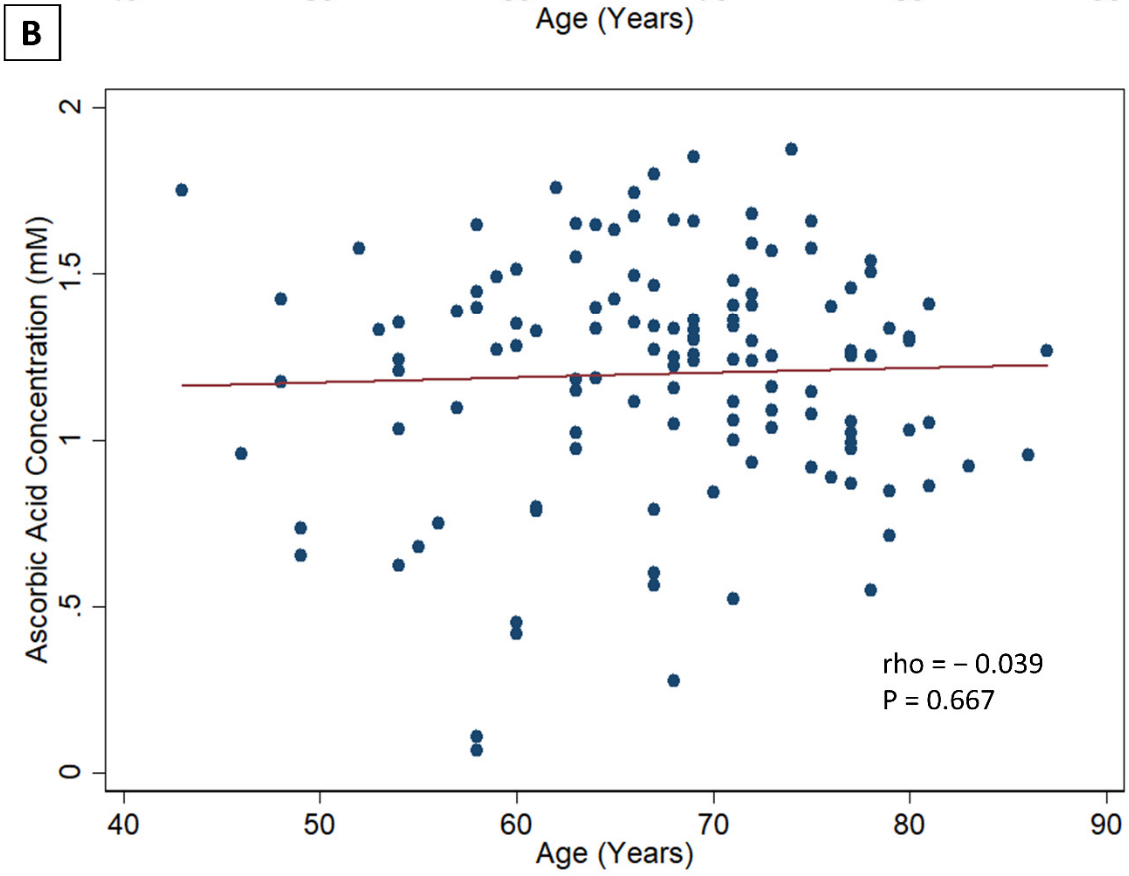

Supplementary Figure S2. Scatter plot showing the correlation between aqueous humor total antioxidant capacity (TAC)/ascorbic acid concentration (AA) and Patient Age: (**A**) There was no significant correlation between aqueous humor TAC and patient age, as measured by Spearman correlation coefficient (P value = 0.524); (**B**) there was no significant correlation between aqueous humor TAC and patient age, as measured by Spearman correlation coefficient (P value = 0.667).
