# Supplementary material for: An Assessment of Cataract Severity Based on Antioxidant Status and Ascorbic Acid Levels in Aqueous Humor
Source: Antioxidants (Basel). 2022 Feb 16;11(2):397. doi: 10.3390/antiox11020397 (PMC8869206; doi:10.3390/antiox11020397)
Supplement: Supplementary file 1 [file antioxidants-11-00397-s001.zip › Supplementary Table S1-edited.pdf]

Table S1. Univariate and multivariate linear regression analyses of ascorbic acid for cumulative dissipated energy during the phacoemulsification process.

	Univariate Regression			Multivariate Regression		
**Variable**	**B**	**SE**	***P* value**	**B**	**SE**	***P* value**
Ascorbic Acid Level	-15.53	5.23	0.004**	-16.27	5.30	0.003**
**Basic characteristics**						
OD / OS	0.31	3.79	0.934	-0.13	3.68	0.972
Age	0.45	0.21	0.034*	0.66	0.28	0.021*
Gender	-2.58	3.77	0.496	1.16	3.81	0.762
BMI	-0.01	0.54	0.99	-0.38	0.58	0.509
Disease diagnosis	-0.11	6.09	0.986	12.50	8.23	0.131
**Underlying disease**						
Hypertension	0.61	3.87	0.876	0.516	4.52	0.909
Diabetes mellitus	11.4	4.48	0.012*	9.319	4.60	0.045
Other underlying systemic disease	-3.73	3.81	0.329	-4.584	4.26	0.284

B = beta coefficient; BMI = body mass index; OD = oculus dextrus; OS = oculus sinister; SE = standard error,

Age, eye site, gender, body mass index, disease diagnosis, and underlying diseases were adjusted in the multivariate regression as confounding factors.

P value of the multivariate regression model is 0.001.

Other systemic diseases include dyslipidemia, heart diseases, kidney diseases, lung diseases, liver diseases, stroke, immunocompromised status, and autoimmune diseases.*: p-value < 0.05; **: p-value < 0.01
