# Supplementary material for: An Assessment of Cataract Severity Based on Antioxidant Status and Ascorbic Acid Levels in Aqueous Humor
Source: Antioxidants (Basel). 2022 Feb 16;11(2):397. doi: 10.3390/antiox11020397 (PMC8869206; doi:10.3390/antiox11020397)
Supplement: Supplementary file 1 [file antioxidants-11-00397-s001.zip › Supplementary Table S2-edited.pdf]

Table S2. Univariate and multivariate linear regression analyses for cumulative dissipated energy during the phacoemulsification process based on nuclear opalescence and cortical cataract.

Variable	Univariate Regression			Multivariate Regression		
	**B**	**SE**	***P* value**	**B**	**SE**	***P* value**
Nuclear Opalescence	1.75	2.719	0.521	0.992	2.776	0.722
Cortical Cataract	−0.212	3.532	0.952	−1.468	3.528	0.678

B = beta coefficient; SE = standard error

Age, eye site, gender, body mass index, disease diagnosis, and underlying diseases were adjusted in the multivariate regression as confounding factors.

P value of the multivariate regression model is 0.286 for the nuclear opalescence model and 0.094 for the cortical cataract model.
