# Supplementary material for: An Assessment of Cataract Severity Based on Antioxidant Status and Ascorbic Acid Levels in Aqueous Humor
Source: Antioxidants (Basel). 2022 Feb 16;11(2):397. doi: 10.3390/antiox11020397 (PMC8869206; doi:10.3390/antiox11020397)
Supplement: Supplementary file 1 [file antioxidants-11-00397-s001.zip › Supplementary Table S3-edited.pdf]

**Table S3.** Univariate and multivariate linear regression analyses for the improvement of best corrected visual acuity after cataract surgery.

| | Univariate Regression | | | Multivariate Regression | | |
|---|---|---|---|---|---|---|
| **Variable** | **B** | **SE** | ***P* value** | **B** | **SE** | ***P* value** |
| Total antioxidant capacity | -0.147 | 0.111 | 0.188 | -0.109 | 0.113 | 0.335 |
| **Basic characteristics** | | | | | | |
| OD / OS | -0.09 | 0.077 | 0.245 | -0.09 | 0.074 | 0.229 |
| Age | -0.013 | 0.004 | 0.002** | -0.009 | 0.006 | 0.107 |
| Gender | 0.053 | 0.077 | 0.497 | 0.038 | 0.076 | 0.619 |
| BMI | 0.004 | 0.011 | 0.72 | 0.001 | 0.011 | 0.961 |
| Disease diagnosis | 0.325 | 0.107 | 0.003** | 0.234 | 0.157 | 0.14 |
| **Underlying disease** | | | | | | |
| Hypertension | -0.008 | 0.078 | 0.92 | 0.056 | 0.091 | 0.543 |
| Diabetes mellitus | 0.138 | 0.098 | 0.163 | 0.203 | 0.101 | 0.048* |
| Other underlying systemic diseases | 0.011 | 0.078 | 0.891 | 0.053 | 0.087 | 0.546 |

B = beta coefficient; BMI = body mass index; OD = oculus dextrus; OS = oculus sinister; SE = standard error

Age, eye site, gender, body mass index, disease diagnosis, and underlying diseases were adjusted in the multivariate regression as confounding factors.

*P* value of the multivariate regression model is 0.02.

Other systemic diseases include dyslipidemia, heart diseases, kidney diseases, lung diseases, liver diseases, stroke, immunocompromised status, and autoimmune diseases.

**: p-value < 0.01
