# Supplementary material for: An Assessment of Cataract Severity Based on Antioxidant Status and Ascorbic Acid Levels in Aqueous Humor
Source: Antioxidants (Basel). 2022 Feb 16;11(2):397. doi: 10.3390/antiox11020397 (PMC8869206; doi:10.3390/antiox11020397)
Supplement: Supplementary file 1 [file antioxidants-11-00397-s001.zip › Supplementary Table S4-edited.pdf]

Table S4. Univariate and multivariate linear regression analyses for changes in intraocular pressure after cataract surgery.

	**Univariate Regression**			**Multivariate Regression**		
**Variable**	**B**	**SE**	***P* value**	**B**	**SE**	***P* value**
Total antioxidant capacity	0.375	0.645	0.562	0.65	0.662	0.327
**Basic characteristics**						
OD/OS	-0.575	0.476	0.23	-0.609	0.479	0.206
Age	0	0.027	0.99	0.042	0.036	0.243
Gender	-0.855	0.471	0.072	-1.123	0.494	0.025
BMI	-0.016	0.069	0.812	-0.04	0.075	0.599
Disease diagnosis	0.943	0.764	0.219	2.161	1.059	0.043*
**Underlying disease**						
Hypertension	0.038	0.488	0.939	0.008	0.582	0.988
Diabetes mellitus	-0.138	0.58	0.812	-0.162	0.601	0.788
Other underlying systemic diseases	0.186	0.482	0.701	0.449	0.545	0.412

B = beta coefficient; BMI = body mass index; OD = oculus dextrus; OS = oculus sinister; SE = standard error

Age, eye site, gender, body mass index, disease diagnosis, and underlying diseases were adjusted in the multivariate regression as confounding factors.

P value of the multivariate regression model is 0.32.

Other systemic diseases include dyslipidemia, heart disease, kidney disease, lung disease, liver disease, stroke, immunocompromised status, and autoimmune disease.

*: p-value < 0.05
